# Survival benefit of palliative gastrectomy followed by chemotherapy in stage IV gastric signet ring cell carcinoma patients: A large population‐based study

**DOI:** 10.1002/cam4.2521

**Published:** 2019-08-25

**Authors:** Tao Shi, Xueru Song, Qin Liu, Yang Yang, Lixia Yu, Baorui Liu, Jia Wei

**Affiliations:** ^1^ The Comprehensive Cancer Centre of Drum Tower Hospital Medical School of Nanjing University and Clinical Cancer Institute of Nanjing University Nanjing China

**Keywords:** chemotherapy, gastric signet ring cell carcinoma, palliative gastrectomy, SEER database, stage IV gastric cancer

## Abstract

**Background:**

Stage IV gastric signet ring cell carcinoma (SRCC) is a type of malignant gastric cancer (GC) with poorer survival compared to metastatic non‐SRCC gastric cancer (NOS). However, chemotherapy alone was unable to maintain long‐term survival. This study aimed to evaluate survival benefit of palliative gastrectomy plus chemotherapy (PG+C) for metastatic gastric SRCC.

**Methods:**

We obtained data on gastric cancer patients between 2010 and 2015 from the Surveillance, Epidemiology, and End Results (SEER) database. Statistical methods included *χ* ^2^ tests, Kaplan‐Meier curves, COX models, propensity score matching (PSM) and subgroup analysis.

**Results:**

Among 27 240 gastric cancer patients included, 4638 (17.03%) were SRCC patients. The proportion of patients with younger age, female gender, poorly differentiated grade and M1 stage was higher in SRCC than in NOS (*P* < .001). Multivariate analysis revealed that multiple metastatic sites (HR = 1.39, 95% CI: 1.14‐1.69, *P* = .001) was associated with increased mortality risk in metastatic SRCC. Median survival time was improved in metastatic SRCC receiving PG+C compared to PG/C alone (13 vs 7 months, *P* < .001). Notably, in subgroup analysis, 13 of 17 groups of metastatic SRCC patients with PG+C had prolonged overall survival compared to chemotherapy alone, especially for those with only one metastatic site (HR = 0.61, 95% CI: 0.51‐0.73, *P* < .001).

**Conclusions:**

Our results suggested that there exists at least a selective group of stage IV gastric SRCC patients, who could benefit from palliative gastrectomy followed by chemotherapy compared to chemotherapy alone. Further prospective trials are needed to support our conclusion.

## Graphical abstract

Stage IV gastric signet ring cell carcinoma (SRCC) is a type of malignant gastric cancer with higher metastasis rate and poorer overall survival compared to non‐SRCC gastric cancer (NOS). There exists at least a selective group of stage IV gastric SRCC patients, who could benefit from palliative gastrectomy followed by chemotherapy compared to chemotherapy alone.

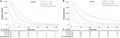

## 1 INTRODUCTION

Gastric cancer is one of the most common malignant tumors of the digestive tract, and currently accounts for 8.2% of all new cancer cases worldwide.^1^ Adenocarcinomas represent the majority of gastric cancers, while signet ring cell carcinoma (SRCC) is a poorly differentiated subtype of gastric carcinoma with unique clinical characteristics and poor survival rates.^2^, ^3^, ^4^ Previous studies indicated that gastric SRCC has different risk factors compared to non‐SRCC gastric cancer (NOS) types, including older age, female gender, smoking, obesity and American Joint Committee on Cancer (AJCC) IV stage.^5^, ^6^, ^7^ Pathologically, gastric SRCC consists of scattered malignant cells containing abundant intracytoplasmic mucin, and is associated with rapid growth and diffuse infiltration of surrounding tissues.^6^, ^8^ Also, previous studies reported that SRCC was not cohesive and prone to invasion of the submucosal and subserosal layers, which also allowed for the increased incidence and poor survival of metastatic SRCC.^9^, ^10^, ^11^

Palliative systemic therapy (mostly chemotherapy) was recommended as standard management for stage IV gastric SRCC.^12^ A multicenter study reported that triplet chemotherapy with docetaxel‐5FU‐oxaliplatin appeared to be effective as first‐line treatment for patients with metastatic or locally advanced non‐resectable gastric SRCC.^13^ However, the role of palliative gastrectomy in stage IV gastric cancer is still controversial. Previous retrospective studies and clinical trials showed that palliative gastrectomy was associated with improved overall survival for metastatic gastric cancer patients.^14^, ^15^, ^16^ Conversely, one phase 3, randomized controlled trial (REGATTA trial) showed that gastrectomy followed by chemotherapy failed to have any survival benefit compared with chemotherapy alone in advanced gastric cancer.^17^ Thus, the appropriate justification for the role of palliative gastrectomy is still needed. Notably, up till now, there is no such analysis focusing on the treatment effect of palliative gastrectomy on stage IV gastric SRCC patients. Considering the higher metastatic rate and the poorer overall survival of gastric SRCC compared to NOS, it is of great necessity to assess the role of palliative gastrectomy followed by adjuvant chemotherapy for stage IV gastric SRCC patients.

Therefore, in this study, the primary aim was to assess the therapeutic effects of palliative gastrectomy and chemotherapy on the survival of stage IV gastric SRCC patients based on a large Western population. We also identified significant clinicopathological characteristics and independent prognosis factors of these patients.

## 2 MATERIALS AND METHODS

### 2.1 Data source

Cases of gastric cancer were identified from the Surveillance, Epidemiology, and End Results (SEER) database. The SEER database is an openly accessed database with information on cancer incidence and survival from 18 population‐based cancer registries, representing approximately 28% of the United States population (http://seer.cancer.gov/ about/overview.html).^18^ SEER is supported by the Surveillance Research Program (SRP) in National Cancer Institute's Division of Cancer Control and Population Sciences (DCCPS). We used the SEER database version available on April 2019 (Noember 2018 Submission). The methods we employed were consistent with the criteria of the SEER database. All TNM classification was restaged according to the criteria described in the American Joint Committee on Cancer (AJCC) Cancer Staging Manual, 8th edition, 2017 (Stages I, II, III, and IV).

### 2.2 Patient selection

**Figure 1:**
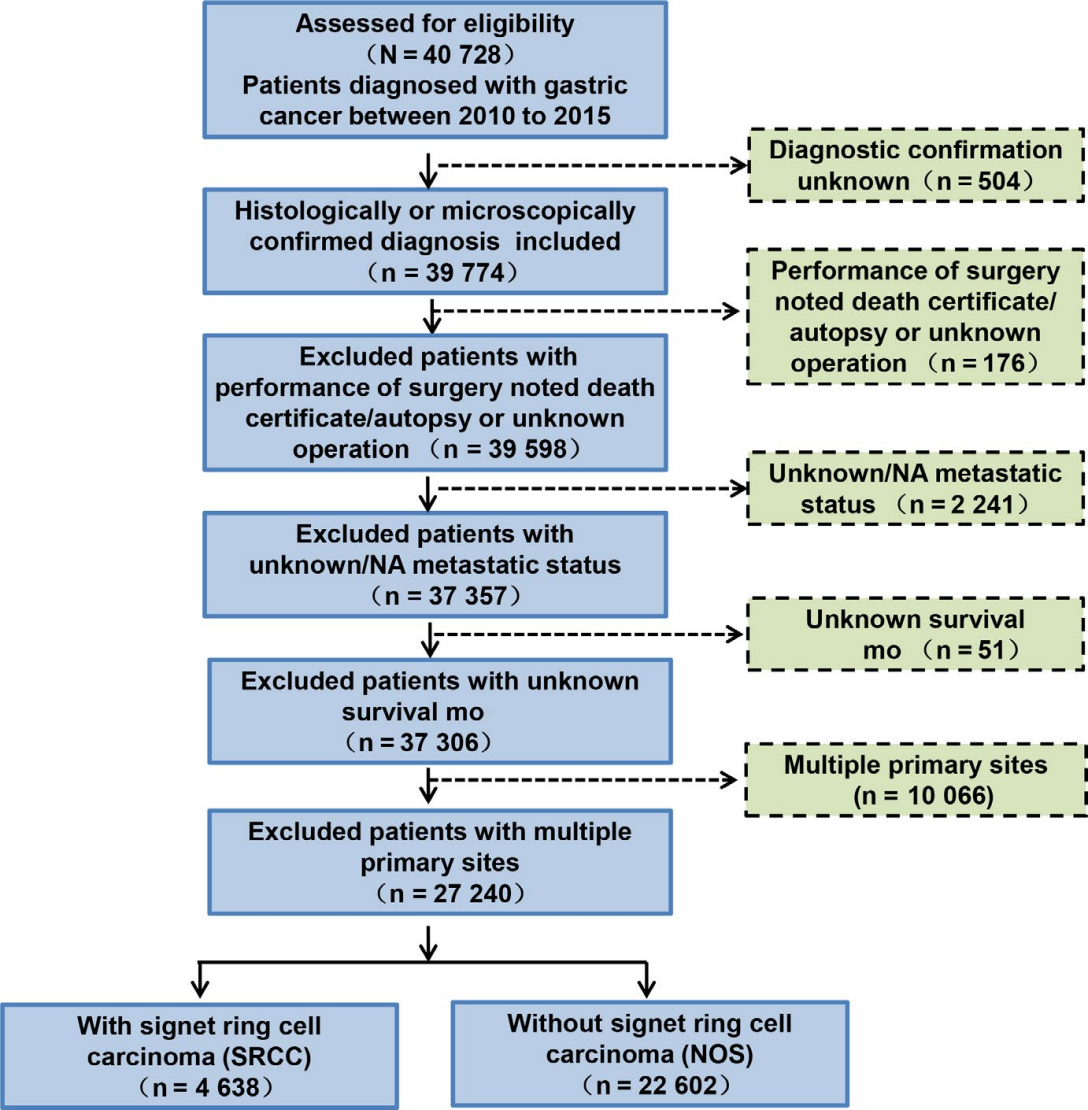
Flow chart of inclusion and exclusion process of the Surveillance, Epidemiology and End Results (SEER) patient dataset

Patient data of gastric cancer, including gastric SRCC from 2010 to 2015 were obtained from the SEER database according to the International Classification of Diseases for Oncology (Third Edition, ICD‐O‐3, SRCC identified as 8490). Patient selection criteria were as follows: (a) confirmation of gastric cancer diagnosis histologically or microscopically; (b) exclusion of patients who had unknown surgeries; (c) exclusion of patients with unknown or absent metastatic status; (d) exclusion of patients with unknown survival months; (e) exclusion of patients with multiple primary tumor sites. As a result, 27 240 patients were assessed for eligibility, including 4638 gastric SRCC patients and 22 602 NOS patients (Figure 1). In subgroup analysis, patients with distant metastasis were identified as AJCC stage IV (M1 stage at diagnosis). Patient clinical variables included age at diagnosis, gender, race, year of diagnosis, tumor grade, AJCC stage, TNM stage at diagnosis, tumor size, tumor location, regional nodes examined, and therapies employed (surgery, chemotherapy, and radiotherapy).

### 2.3 Statistical analysis

The R version 3.5.0 (http://www.R-project.org/) was employed for all statistical analysis. Chi‐square tests (*χ* ^2^ test) were used to compare clinicopathological characteristics between gastric SRCC and NOS patients. The overall survival (OS) was the primary endpoint outcome defined from the date of operation to the date of death or the latest follow‐up. The 5‐year OS rates were the ratio of patients alive after five years from the operation date among all patients included. Kaplan‐Meier curves and log‐rank tests were used to draw overall survival curves within different patient subgroups. Also, to analyze the prognostic factors of stage IV gastric SRCC and NOS patients, we employed univariate and multivariate Cox regression models to estimate HR (hazard ratio) and exact 95% CIs (confidence intervals). Propensity score matching (PSM) was used to adjust numerical differences between gastric SRCC and NOS patients. The forest plot was used to compare the impact of palliative gastrectomy followed by chemotherapy versus palliative gastrectomy or chemotherapy alone among different metastatic gastric SRCC subgroups. All statistical tests were performed two‐sided and *P* values <.05 were considered to be statistically significant.

## 3 RESULTS

### 3.1 Patient characteristics and overall survival of gastric NOS and SRCC

Data from a total of 27 240 eligible gastric cancer patients diagnosed between 2010 and 2015 were obtained from the SEER database. Among these, 4638 were gastric SRCC patients (17.03%) and 22 602 were NOS patients (82.97%, Table 1). In this cohort, 2701 SRCC patients (58.24%) were younger than 65 years while 9669 NOS patients (42.78%) were younger than 65, indicating that patients diagnosed with SRCC present a younger age compared with other types of gastric cancer (*P* < .001). Regarding gender, more female patients were diagnosed with SRCC (47.26%) compared with NOS patients (37%) (*P* < .001). Additionally, compared with NOS patients, patients with SRCC are more likely to have a poorly differentiated tumor grade (77.19% vs 43.01%, *P* < .001), III/IV AJCC stage (21.07% vs 17.58% in III, 43.31% vs 34.08% in Ⅳ, *P* < .001) and T4 stage (25.61% vs 16.82%, *P* < .001), which were in accordance with the pathological features of gastric signet ring cell carcinoma reported previously.^2^ Importantly, more SRCC patients were in M1 stage than NOS patients (42.93% vs 33.76%, *P* < .001). With regards to treatment, the proportion of surgery or radiotherapy received was similar between SRCC and NOS patients (41.29% vs 47.82% in surgery, *P* < .001; 14.6% vs 13.19% in radiotherapy, *P* = .011), However, more SRCC patients chose to receive chemotherapy compared with NOS patients (60.69% vs 49.78%, *P* < .001). Additional cohort information is shown in Table 1. In order to eliminate the impact from the difference in the number of SRCC and NOS patients, we also conducted propensity score matching (PSM) to analyze and adjust patient characteristics for gender, race, and age (Table 1). Clinicopathological results between SRCC and NOS patients were essentially the same following PSM. We also analyzed patient characteristics of stage IV gastric SRCC and NOS in Table S1 and the clinicopathological differences were basically very similar with results in Table 1.

**Table 1:** Clinicopathological Characteristics of gastric NOS and SRCC Patients

Variable	NOS n = 22 602 (%)	SRCC n = 4638 (%)	*P*	NOS PSM n = 4638 (%)	SRCC PSM n = 4638 (%)	*P*
Age (y)						
<65	9669 (42.78)	2701 (58.24)		2701 (58.24)	2701 (58.24)	
≥65	12 933 (57.22)	1937 (41.76)	<.001	1937 (41.76)	1937 (41.76)	1
Gender						
Male	14 239 (63)	2446 (52.74)		2446 (52.74)	2446 (52.74)	
Female	8363 (37)	2192 (47.26)	<.001	2192 (47.26)	2192 (47.26)	1
Race						
White	15 820 (69.99)	3254 (70.16)		3254 (70.16)	3254 (70.16)	
Black	3270 (14.47)	571 (12.31)		571 (12.31)	571 (12.31)	
Other	3512 (15.54)	813 (17.53)	<.001	813 (17.53)	813 (17.53)	1
Year						
2010	3572 (15.8)	731 (15.76)		773 (16.67)	731 (15.76)	
2011	3548 (15.7)	740 (15.96)		714 (15.39)	740 (15.96)	
2012	3780 (16.72)	802 (17.29)		774 (16.69)	802 (17.29)	
2013	3845 (17.01)	746 (16.08)		802 (17.29)	746 (16.08)	
2014	3969 (17.56)	823 (17.74)		797 (17.18)	823 (17.74)	
2015	3888 (17.2)	796 (17.16)	.708	778 (16.77)	796 (17.16)	.443
Tumor grade						
Well	2045 (9.05)	6 (0.13)		476 (10.26)	6 (0.13)	
Moderately	5544 (24.53)	86 (1.85)		1070 (23.07)	86 (1.85)	
Poorly	9722 (43.01)	3580 (77.19)		2008 (43.29)	3580 (77.19)	
Undifferentiated	470 (2.08)	91 (1.96)		104 (2.24)	91 (1.96)	
Unknown	4821 (21.33)	875 (18.87)	<.001	980 (21.13)	875 (18.87)	<.001
Tumor location						
Upper stomach	8648 (38.26)	967 (20.85)		1694 (36.52)	967 (20.85)	
Middle stomach	4822 (21.33)	1215 (26.2)		1009 (21.76)	1215 (26.2)	
Lower stomach	4303 (19.04)	1089 (23.48)		950 (20.48)	1089 (23.48)	
Overlapping	1507 (6.67)	530 (11.43)		287 (6.19)	530 (11.43)	
Stomach NOS	3322 (14.7)	837 (18.05)	<.001	698 (15.05)	837 (18.05)	<.001
AJCC						
0,I,II	8502 (34.55)	1341 (26.7)		1720 (34.25)	1341 (26.7)	
III	4327 (17.58)	1058 (21.07)		847 (16.87)	1058 (21.07)	
IV	8386 (34.08)	2175 (43.31)		1775 (35.34)	2175 (43.31)	
Unknown	3393 (13.79)	448 (8.92)	<.001	680 (13.54)	448 (8.92)	<.001
T‐stage						
Tis,T1,T2	8293 (36.69)	1375 (29.65)		2454 (52.91)	2102 (45.32)	
T3	5502 (24.34)	1049 (22.62)		1505 (32.45)	1504 (32.43)	
T4	3801 (16.82)	1188 (25.61)		316 (6.81)	626 (13.5)	
Unknown	5006 (22.15)	1026 (22.12)	<.001	363 (7.83)	406 (8.75)	<.001
N‐stage						
N0	11 699 (51.76)	2102 (45.32)		2454 (52.91)	2102 (45.32)	
N1,N2	7470 (33.05)	1504 (32.43)		1505 (32.45)	1504 (32.43)	
N3	1613 (7.14)	626 (13.5)		316 (6.81)	626 (13.5)	
Unknown	1820 (8.05)	406 (8.75)	<.001	363 (7.83)	406 (8.75)	<.001
M‐stage						
M0	14 971 (66.24)	2647 (57.07)		2999 (64.66)	2647 (57.07)	
M1	7631 (33.76)	1991 (42.93)	<.001	1639 (35.34)	1991 (42.93)	<.001
Regional lymph nodes						
None	13 998 (61.93)	2691 (58.02)		2904 (62.61)	2691 (58.02)	
Negative	3868 (17.11)	670 (14.45)		795 (17.14)	670 (14.45)	
Positive	4425 (19.58)	1233 (26.58)		881 (19)	1233 (26.58)	
Unknown	311 (1.38)	44 (0.95)	<.001	58 (1.25)	44 (0.95)	<.001
Surgery						
No	11 794 (52.18)	2723 (58.71)		2389 (51.51)	2723 (58.71)	
Yes	10 808 (47.82)	1915 (41.29)	<.001	2249 (48.49)	1915 (41.29)	<.001
Radiotherapy						
No	19 620 (86.81)	3961 (85.4)		3990 (86.03)	3961 (85.4)	
Yes	2982 (13.19)	677 (14.6)	.011	648 (13.97)	677 (14.6)	.406
Chemotherapy						
No/Unknown	11 631 (51.46)	1823 (39.31)		2329 (50.22)	1823 (39.31)	
Yes	10 971 (48.54)	2815 (60.69)	<.001	2309 (49.78)	2815 (60.69)	<.001

Abbreviations: NOS, non‐SRCC gastric cancer; PSM, propensity score matching; SRCC, gastric signet ring cell carcinoma.

Additionally, considering the high rate of metastasis in gastric SRCC patients (Table 1), we further analyzed the five‐year overall survival (OS) among gastric SRCC and NOS groups with or without distant metastasis. The survival results showed that SRCC type and distant metastasis were associated with poorer survival (Figure S1A, *P* < .001). M1 stage SRCC patients had the worst survival, whose 3‐year OS was 1.76% compared to 4.38% (M1 NOS), 22.18% (M0 SRCC) and 30.71% (M0 NOS). Survival outcome between these subgroups was essentially the same after PSM (Figure S1B).

### 3.2 Patient characteristics of stage IV gastric SRCC with different treatment

Considering the higher metastatic rate and poorer survival of stage IV gastric SRCC compared with NOS, we made further analysis on these patients with different treatments including palliative gastrectomy plus chemotherapy (PG+C), palliative gastrectomy or chemotherapy alone (PG/C) and no treatment (Table 2). T and N stage were not included in the analysis of stage IV patients due to the low accuracy of staging among patients who did not undergo surgery. The number of patients under age 65 was larger in PG+C (77.22%) and PG/C groups (74.01%) than no treatment group (51.31%) (*P* < .001). Also, the proportion of stage IV gastric SRCC patients with poorly differentiated grade or tumor at overlapping location was higher in PG+C (80%; 21.67%) and PG/C (74.1%; 14.03%) groups than in the no treatment group (62.87%; 9.86%) (*P* < .001). In addition, more patients with only one metastatic site received PG+C (95.56%) compared to patients with PG/C (82.53%) or no treatment (83.2%) (*P* < .001). Other clinicopathological differences among these three groups are also shown in Table 2.

**Table 2:** Clinicopathological Characteristics of stage IV SRCC Patients with PG+C, PG/C or with no treatment

Variable	PG+C n = 180 (%)	PG/C n = 1162 (%)	No treatment n = 649 (%)	*P* value
Age (y)				
<65	139 (77.22)	860 (74.01)	333 (51.31)	
≥65	41 (22.78)	302 (25.99)	316 (48.69)	<.001
Gender				
Male	82 (45.56)	605 (52.07)	350 (53.93)	
Female	98 (54.44)	557 (47.93)	299 (46.07)	.138
Race				
White	138 (76.67)	838 (72.12)	464 (71.49)	
Black	11 (6.11)	139 (11.96)	89 (13.71)	
Other	31 (17.22)	185 (15.92)	96 (14.79)	.093
Year				
2010	42 (23.33)	162 (13.94)	104 (16.02)	
2011	26 (14.44)	170 (14.63)	97 (14.95)	
2012	34 (18.89)	188 (16.18)	102 (15.72)	
2013	25 (13.89)	183 (15.75)	114 (17.57)	
2014	30 (16.67)	232 (19.97)	112 (17.26)	
2015	23 (12.78)	227 (19.54)	120 (18.49)	.065
Tumor grade				
Well/ Moderately	5 (2.78)	15 (1.29)	12 (1.85)	
Poorly	144 (80)	861 (74.1)	408 (62.87)	
Undifferentiated	6 (3.33)	10 (0.86)	11 (1.69)	
Unknown	25 (13.89)	276 (23.75)	218 (33.59)	<.001
Tumor location				
Upper stomach	14 (7.78)	246 (21.17)	116 (17.87)	
Middle stomach	51 (28.33)	304 (26.16)	151 (23.27)	
Lower stomach	51 (28.33)	190 (16.35)	99 (15.25)	
Overlapping	39 (21.67)	163 (14.03)	64 (9.86)	
Stomach NOS	25 (13.89)	259 (22.29)	219 (33.74)	<.001
Distant metastasis				
One site	172 (95.56)	959 (82.53)	540 (83.2)	
Multiple sites	8 (4.44)	203 (17.47)	109 (16.8)	<.001
Surgery				
No	0 (0.00)	1092 (93.98)	649 (100.00)	
Yes	180 (100.00)	70 (6.02)	0 (0.00)	
Chemotherapy				
No/Unknown	0 (0.00)	70 (6.02)	649 (100.00)	
Yes	180 (100.00)	1092 (93.98)	0 (0.00)	

### 3.3 Identifying prognosis factors for patients with stage IV gastric SRCC

To further explore the risk factors related to long‐term survival outcome of metastatic gastric SRCC, we employed univariate and multivariate Cox regression analyses to identify protective or adverse prognostic factors. T and N stage were not included in the analysis of stage IV patients due to the low accuracy of staging among these patients. As shown in Table 3, results of multivariate Cox regression suggested that among metastatic gastric SRCC patients, palliative gastrectomy (HR = 0.66, 95% CI: 0.51‐0.85, *P* < .001) and chemotherapy (HR = 0.28, 95% CI: 0.24‐0.32, *P* < .001) were considered as protective prognosis factors, while multiple metastatic sites (HR = 1.39, 95% CI: 1.14‐1.69, *P* = .001) was an adverse prognosis factor. Other independent prognosis factors of stage IV gastric SRCC identified in the univariate Cox regression are listed in Table 3.

**Table 3:** Univariate and multivariate analyses for stage IV gastric SRCC Patients

Variable	Univariable			Multivariable		
	HR	95% CI	*P* value	HR	95% CI	*P* value
Age (y)						
≦65	Ref			Ref		
>65	1.38	1.26‐1.52	<.001	1.08	0.94‐1.23	.285
Gender						
Male	Ref					
Female	0.88	0.8‐0.96	.006	0.95	0.84‐1.08	.458
Race						
White	Ref					
Black	1.11	0.96‐1.28	.155			
Other	0.96	0.84‐1.09	.488			
Tumor grade						
Well	Ref					
Moderately	1.06	0.25‐4.44	.938			
Poorly	1	0.25‐4	.998			
Undifferentiated	1.1	0.26‐4.63	.901			
Unknown	1.06	0.25‐4.44	.938			
Distant metastasis						
One site	Ref					
Multiple sites	1.38	1.22‐1.57	0	1.39	1.14‐1.69	.001
Primary tumor location						
Upper stomach	Ref			Ref		
Middle stomach	0.93	0.81‐1.07	.294	1.08	0.9‐1.31	.418
Lower stomach	0.98	0.84‐1.14	.785	1.01	0.82‐1.23	.958
Overlapping	0.96	0.82‐1.13	.645	1.13	0.92‐1.39	.249
Stomach NOS	1.28	1.12‐1.47	0	1.25	1.02‐1.53	.029
Palliative gastrectomy						
No	Ref			Ref		
Yes	0.53	0.46‐0.61	<.001	0.66	0.51‐0.85	0
Radiotherapy						
No	Ref			Ref		
Yes	0.56	0.42‐0.74	<.001	1.08	0.76‐1.53	.666
Chemotherapy						
No/Unknown	Ref			Ref		
Yes	0.31	0.28‐0.34	<.001	0.28	0.24‐0.32	0

Abbreviations: NOS, Non‐SRCC gastric cancer; SRCC, Gastric signet ring cell carcinoma.

### 3.4 Survival benefits of palliative gastrectomy plus chemotherapy for stage IV gastric SRCC

Given that assessing treatment effects for metastatic gastric SRCC patients was essential, we next focused on therapeutic benefits of overall survival for these patients. Treatment managements of metastatic gastric SRCC were divided into three groups (PG+C, n = 180; PG/C, n = 1162; no treatment, n = 649). Radiotherapy was not included in further analysis due to the small patient number (n = 55, *P* = .355, Table S1) and it was not found to be an independent prognosis factor in multivariate Cox regression (*P* = .589; Table 3). The findings were as follows.

**Figure 2:**
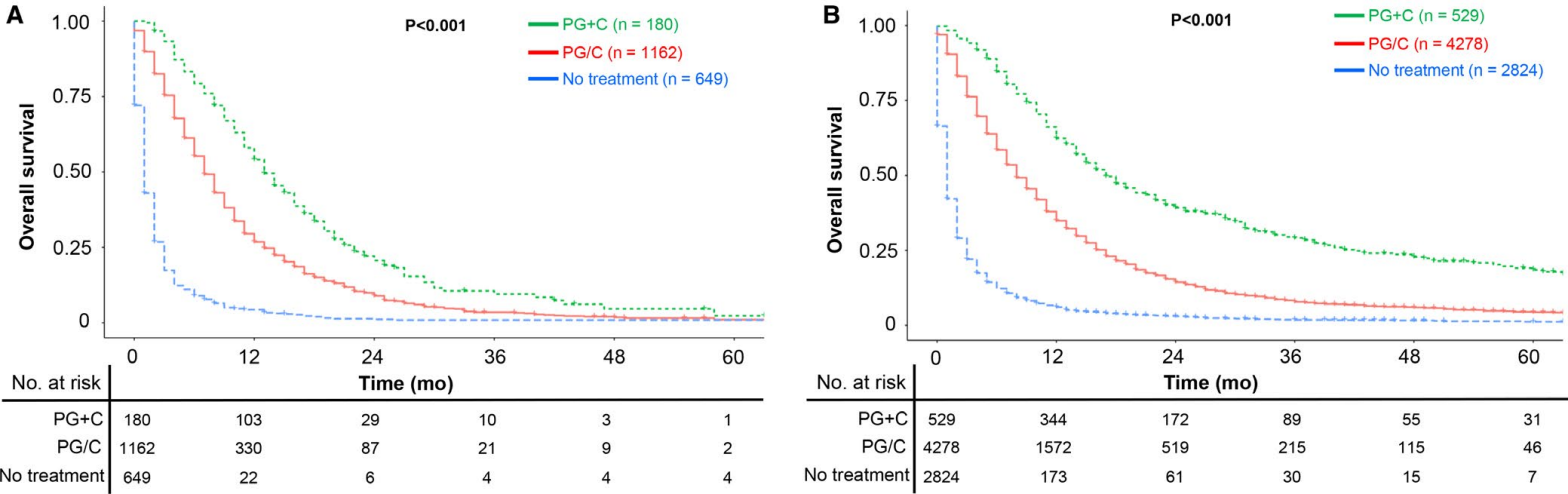
A, OS among stage IV gastric SRCC patients with PG+C, PG/C or with no treatment, *P* < .001; (B) OS among stage IV gastric NOS patients with PG+C, PG/C or with no treatment, *P* < .001; NOS: Non‐specific gastric cancer except SRCC; SRCC: Signet ring cell carcinoma; PG+C: Palliative gastrectomy followed by chemotherapy; PG/C: Palliative gastrectomy or chemotherapy alone

**Figure 3:**
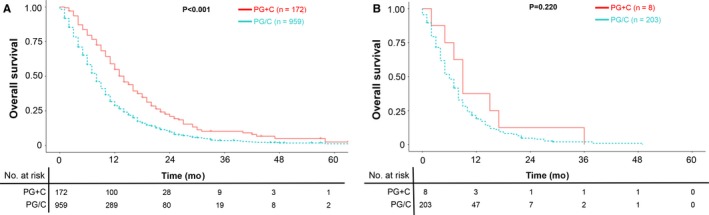
A, OS of PG+C or PG/C among stage IV gastric SRCC patients with only one metastatic site, *P* < .001; (B) OS of PG + C or PG/C among stage IV gastric SRCC patients with multiple metastatic sites, *P* = .220. SRCC: Signet ring cell carcinoma; PG+C: Palliative gastrectomy followed by chemotherapy; PG/C: Palliative gastrectomy or chemotherapy alone

In Figure 2A, median survival time of metastatic SRCC patients receiving PG+C was 13 months (95% CI: 12‐16 months), while the PG/C group was 7 months (95% CI: 7‐8 months). Similar results could also be found among NOS groups in Figure 2B, with 17 months (95% CI: 16‐20 months) of median survival time in PG+C group and 8 months (95% CI: 8‐9 months) in PG/C group. These results indicated that both in metastatic gastric SRCC and NOS, palliative gastrectomy followed by chemotherapy could lead to prolonged overall survival compared to palliative gastrectomy or chemotherapy alone. Notably, when combining these results together, we found that metastatic SRCC receiving PG+C had better overall survival than metastatic NOS receiving PG/C alone (median survival time: 13 vs 8 months, *P* < .001, Figure S2). Moreover, with regards to the number of metastatic sites, PG+C lead to improved overall survival than PG/C among stage IV gastric SRCC patients with only one metastatic site (*P* < .001, Figure 3A), while these two groups had no statistical difference among patients with multiple metastatic sites (*P* = .220, Figure 3B).

Considering the poorer prognosis of gastric SRCC compared with NOS, these findings emphasized the value of palliative gastrectomy plus chemotherapy in stage IV gastric SRCC treatment, especially those with only one metastatic site.

### 3.5 Subgroup analysis of stage IV gastric SRCC patients

**Figure 4:**
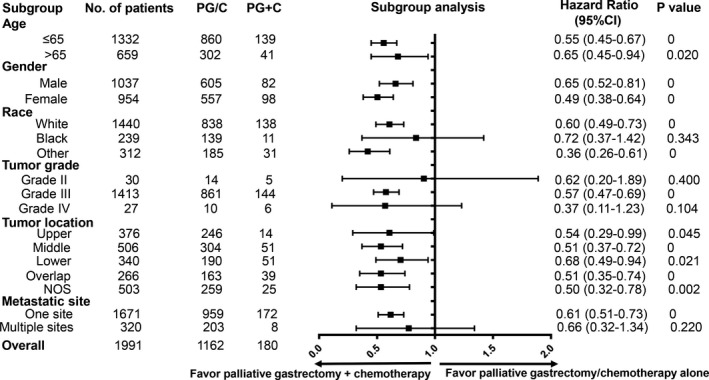
Subgroup analysis of PG+C and PG/C among stage IV gastric SRCC patients. SRCC: Signet ring cell carcinoma; PG+C: Palliative gastrectomy followed by chemotherapy; PG/C: Palliative gastrectomy or chemotherapy alone. Grade II: Moderately differentiated grade, Grade III: Poorly differentiated grade, Grade IV: Undifferentiated grade

After finding the prolonged survival of palliative gastrectomy plus chemotherapy compared to palliative gastrectomy or chemotherapy alone in metastatic gastric SRCC, we next aimed to analyze the prognostic consistency between these two treatment strategies. Metastatic SRCC patients were divided into subgroups based on the clinicopathological characteristics identified in Table 3. Cox's regression model was separately used to estimate HR and 95% CI in each subgroup (Figure 4). The results suggested that generally metastatic gastric SRCC patients who underwent PG+C could obtain much more survival benefits than patients who underwent PG/C alone (*P* < .05 arrived in 13 subgroups). Especially, metastatic SRCC patients with only one metastatic site benefited much from PG+C compared to PG/C alone (HR = 0.61, 95% CI: 0.51‐0.73, *P* = 0) while the survival benefit of PG+C was not statistically significant in patients with multiple metastatic sites (*P* = .220). Similar results are also presented in Figure 3. Therefore, it could be more meaningful to implement palliative gastrectomy plus chemotherapy in M1 gastric SRCC patients with only one distant metastatic site.

Taken together, results from subgroup analysis indicated that for metastatic gastric SRCC, there existed as least a selective subgroup of patients, who could obtain survival benefit from palliative gastrectomy plus chemotherapy.

## 4 DISCUSSION

In this study, we analyzed data from 27 240 gastric cancer patients, including 4638 gastric SRCC patients, from the SEER database. To the best of our knowledge, this is the first large population‐based study investigating the prognostic value of palliative gastrectomy followed by chemotherapy among stage IV gastric SRCC patients. The major findings were that in stage IV gastric SRCC, there existed at least a selective group of patients who could have prolonged overall survival with palliative gastrectomy plus chemotherapy compared to chemotherapy alone.

Stage IV gastric signet ring cell carcinoma has long been considered to have worse survival rates compared with other types of adenocarcinoma.^6^, ^19^ Previous studies reported that gastric SRCC had high proportion of patients with poorly differentiated grade, younger age and female gender,^6^, ^7^, ^20^, ^21^, ^22^ which was consistent with the clinicopathological characteristics identified in our study. As to metastasis, the mechanism behind the formation of signet ring cell carcinoma may contribute to its unique features. It has been shown that the activation of PI3K (Phosphoinositide 3‐kinase) through the ErbB2 (Her2)/ ErbB3 (Her3) pathway in signet ring cells could enhance mucin secretion.^23^ Additionally, the adherent junction is disrupted via activation of the Mitogen‐activated protein kinase 1 (MEK1) pathway, which leads to the loss of cell‐cell contact.^24^ Further, it has been reported that signet ring cells were more likely to have transcoelomic metastasis than other gastric cancer cells.^2^, ^25^ These findings could explain the high frequency of metastasis and reoccurrence in gastric SRCC. In our study, the proportion of gastric cancer patients with distant metastasis was significantly higher in SRCC than in NOS (43.31% vs 34.08%, *P* < .001), which was consistent with the pathological features of SRCC reported by previous studies.^23^, ^24^, ^25^ However, further clinical and genetic analyses are still needed to establish improved therapeutic management of these stage IV patients.

Chemotherapy has been the standard palliative management for patients with metastatic gastric cancer.^12^ However, chemotherapy alone failed to maintain the long‐term survival in some part of stage IV gastric cancer patients.^11^, ^26^ The role of palliative gastrectomy for metastatic gastric cancer (mGC) had been assessed in several studies. The first evidence which proved that palliative gastrectomy could bring survival benefit for mGC patients was a Western retrospective analysis from the Dutch Gastric Cancer Trial in 2002.^16^ In this trial they included 285 mGC patients with incurable tumors and found that patients with palliative gastrectomy performed obtained prolonged overall survival than those without primary tumor resection (8.1 vs 5.4 months; *P* < .001). Similarly, a retrospective study in the East achieved the same conclusion in 2012.^27^ Results from this study suggested that palliative gastrectomy could improve the overall survival of patients with late‐stage gastric cancer who could not undergo radical surgery. However, the REGATTA trial proposed the opposite view.^17^ In this phase 3, randomized controlled trial, 89 advanced gastric cancer patients who received gastrectomy followed by chemotherapy failed to have longer median survival time compared to other 86 patients with chemotherapy alone (14.3 vs 16.6 months, one‐sided *P* = .70), indicating the low value of adding palliative gastrectomy to chemotherapy. As a result of these studies, the role of palliative gastrectomy is still controversial in mGC patients, not to mention that until now no research assesses the role of palliative gastrectomy in stage IV gastric SRCC patients.

In our study, palliative gastrectomy was proved as an independently favorable prognostic factor for metastatic gastric SRCC patients. In subgroup analysis, 13 of 17 subgroups of metastatic SRCC obtained survival benefit from PG+C compared to PG/C alone, especially for those with only one metastatic site. Some of previous studies suggested that SRCC is less chemo‐sensitive than non‐SRCC cancers,^28^, ^29^, ^30^, ^31^ so the value of palliative gastrectomy plus chemotherapy may be even higher in stage IV gastric SRCC than NOS. In addition, several recently published studies proved that improved long‐term survival was observed among stage IV GC patients who underwent conversion surgery (primary tumor resection performed in initially unresectable metastatic cancer after responding to chemotherapy).^32^, ^33^, ^34^, ^35^ This also cast light on the therapeutic role of palliative surgery strategies in stage IV gastric SRCC patients. Stage IV gastric cancer patients who were able to undergo surgery could have advantages over other mGC patients in terms of physical and disease conditions, which may account for the improved OS in these patients treated by surgery. Taken results from our study together, palliative gastrectomy is recommended to improve OS for stage IV gastric SRCC patients who have the potential opportunity to undergo surgery.

Although this study had strengths including the large sample size, subgroup analysis and PSM test, some limitations should also be explained. First, metastasis to peritoneum was not assessed in this study due to the absence of peritoneal metastasis data of gastric cancer patients in the SEER database. Thus, the influence of peritoneal metastasis among stage IV gastric SRCC should be further analyzed. Second, the SEER registry does not include detailed information concerning the dose, toxicity, or duration of chemotherapy, so we were not able to further analyze the effects of varying chemotherapy approaches. Finally, research bias could exist in this retrospective study; so the results of this study needed to be validated by future prospective trials.

## 5 CONCLUSION

Stage IV gastric SRCC is a type of malignant gastric cancer with higher metastasis rate and poorer overall survival compared to NOS. Results from our study suggest that there exists at least a selective group of metastatic gastric SRCC patients, who could benefit from palliative gastrectomy followed by chemotherapy compared to chemotherapy alone. Therefore, palliative gastrectomy is recommended for metastatic gastric SRCC patients who have the potential opportunity to undergo palliative surgery. However, further prospective trials are still needed to validate our results so that palliative gastrectomy could be cautiously considered into the management of metastatic gastric SRCC patients.

## CONFLICT OF INTEREST

The authors declare that they have no conflict of interest.

## AUTHOR CONTRIBUTIONS

All authors read and approved the final manuscript. Conception/Design: Jia Wei; Collection and/or assembly of data: All authors; Data analysis and interpretation: All authors; Manuscript writing: Tao Shi; Review and editing, All authors; Final approval of manuscript: All authors.
