# Supplementary figures and images for: Survival benefit of palliative gastrectomy followed by chemotherapy in stage IV gastric signet ring cell carcinoma patients: A large population‐based study
Source: Cancer Med. 2019 Aug 25;8(13):6010–20. doi: 10.1002/cam4.2521 (PMC6792481; doi:10.1002/cam4.2521)

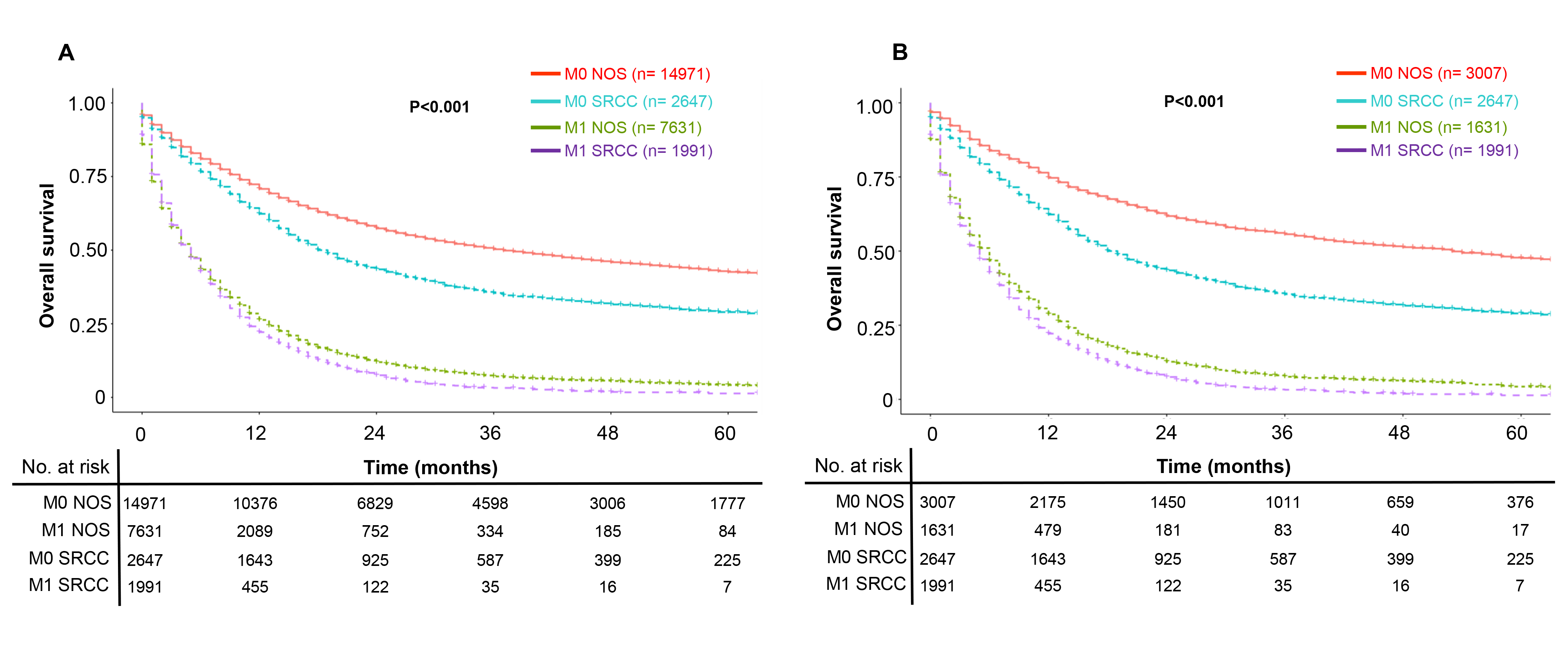

Supplement: Supplementary file 1 [file CAM4-8-6010-s001.tif]

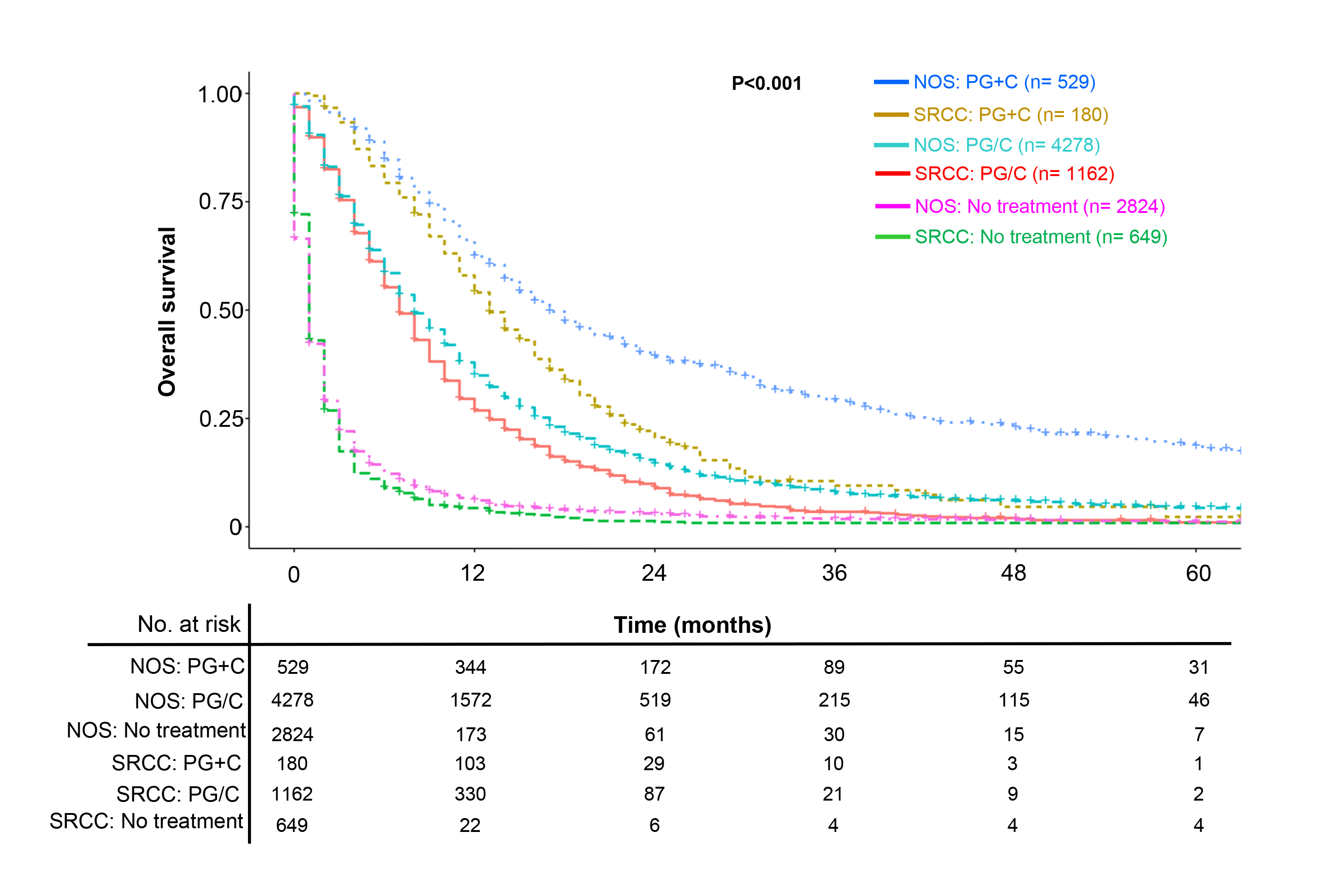

Supplement: Supplementary file 2 [file CAM4-8-6010-s002.tif]
