# Supplementary material for: Survival benefit of palliative gastrectomy followed by chemotherapy in stage IV gastric signet ring cell carcinoma patients: A large population‐based study
Source: Cancer Med. 2019 Aug 25;8(13):6010–20. doi: 10.1002/cam4.2521 (PMC6792481; doi:10.1002/cam4.2521)
Supplement: Supplementary file 3 [file CAM4-8-6010-s003.docx]

**Supplemental table 1.** Clinicopathological Characteristics of M1 NOS and SRCC Patients

| **Variable** | **NOS**  **n=22602 (%)** | **SRCC**  **n=4638 (%)** | ***p*** | **NOS PSM**  **n=1991(%)** | **SRCC PSM**  **n=1991(%)** | ***p*** |
| --- | --- | --- | --- | --- | --- | --- |
| **Age（years）** |  |  |  |  |  |  |
| **<65** | **3706(48.57)** | **1332(66.9)** |  | 1332(66.9) | 1332(66.9) |  |
| ≥65 | 3925(51.43) | 659(33.1) | ﹤0.001 | 659(33.1) | 659(33.1) | 1 |
| **Gender** |  |  |  |  |  |  |
| Male | 5143(67.4) | 1037(52.08) |  | 1037(52.08) | 1037(52.08) |  |
| **Female** | **2488(32.6)** | **954(47.92)** | **﹤0.001** | 954(47.92) | 954(47.92) | 1 |
| **Race** |  |  |  |  |  |  |
| White | 5443(71.33) | 1440(72.33) |  | 1440(72.33) | 1440(72.33) |  |
| Black | 1117(14.64) | 239(12) |  | 239(12) | 239(12) |  |
| Other | 1071(14.03) | 312(15.67) | p=0.004 | 312(15.67) | 312(15.67) | 1 |
| **Year** |  |  |  |  |  |  |
| 2010 | 1223(16.03) | 308(15.47) |  | 325(16.32) | 308(15.47) |  |
| 2011 | 1158(15.17) | 293(14.72) |  | 285(14.31) | 293(14.72) |  |
| 2012 | 1271(16.66) | 324(16.27) |  | 335(16.83) | 324(16.27) |  |
| 2013 | 1299(17.02) | 322(16.17) |  | 333(16.73) | 322(16.17) |  |
| 2014 | 1341(17.57) | 374(18.78) |  | 353(17.73) | 374(18.78) |  |
| 2015 | 1339(17.55) | 370(18.58) | p=0.608 | 360(18.08) | 370(18.58) | p=0.892 |
| **Tumor grade** |  |  |  |  |  |  |
| Well | 180(2.36) | 2(0.1) |  | 46(2.31) | 2(0.1) |  |
| Moderately | 1593(20.88) | 30(1.51) |  | 368(18.48) | 30(1.51) |  |
| **Poorly** | **3861(50.6)** | **1413(70.97)** |  | **1046(52.54)** | **1413(70.97)** |  |
| Undifferentiated | 151(1.98) | 27(1.36) |  | 31(1.56) | 27(1.36) |  |
| Unknown | 1846(24.19) | 519(26.07) | ﹤0.001 | 500(25.11) | 519(26.07) | ﹤0.001 |
| **Tumor location** |  |  |  |  |  |  |
| Upper stomach | 3076(40.31) | 376(18.88) |  | 758(38.07) | 376(18.88) |  |
| **Middle stomach** | **1341(17.57)** | **506(25.41)** |  | **358(17.98)** | **506(25.41)** |  |
| Lower stomach | 1189(15.58) | 340(17.08) |  | 325(16.32) | 340(17.08) |  |
| **Overlapping** | **620(8.12)** | **266(13.36)** |  | **156(7.84)** | **266(13.36)** |  |
| Stomach NOS | 1405(18.41) | 503(25.26) | ﹤0.001 | 394(19.79) | 503(25.26) | ﹤0.001 |
| **Distant metastasis** |  |  |  |  |  |  |
| **One site** | **5630(73.78)** | **1671(83.93)** |  | **1464(73.53)** | **1671(83.93)** |  |
| Multiple sites | 2001(26.22) | 320(16.07) | ﹤0.001 | 527(26.47) | 320(16.07) | ﹤0.001 |
| **Surgery** |  |  |  |  |  |  |
| No | 6739(88.31) | 1741(87.44) |  | 1742(87.49) | 1741(87.44) |  |
| Yes | 892(11.69) | 250(12.56) | p=0.305 | 249(12.51) | 250(12.56) | p=1 |
| **Radiotherapy** |  |  |  |  |  |  |
| No | 7394(96.89) | 1936(97.24) |  | 1925(96.69) | 1936(97.24) |  |
| Yes | 237(3.11) | 55(2.76) | p=0.470 | 66(3.31) | 55(2.76) | p=0.355 |
| **Chemotherapy** |  |  |  |  |  |  |
| No/Unknown | 3187(41.76) | 719(36.11) |  | 774(38.87) | 719(36.11) |  |
| **Yes** | **4444(58.24)** | **1272(63.89)** | **﹤0.001** | 1217(61.13) | 1272(63.89) | p=0.077 |

† NOS: Non-SRCC gastric cancer; SRCC: Gastric signet ring cell carcinoma; PSM: Propensity score matching
